# Using Aiptasia as a Model to Study Metabolic Interactions in Cnidarian-*Symbiodinium* Symbioses

**DOI:** 10.3389/fphys.2018.00214

**Published:** 2018-03-16

**Authors:** Nils Rädecker, Jean-Baptiste Raina, Mathieu Pernice, Gabriela Perna, Paul Guagliardo, Matt R. Kilburn, Manuel Aranda, Christian R. Voolstra

**Affiliations:** ^1^Red Sea Research Center, Division of Biological and Environmental Science and Engineering (BESE), King Abdullah University of Science and Technology (KAUST), Thuwal, Saudi Arabia; ^2^Climate Change Cluster, University of Technology Sydney, Sydney, NSW, Australia; ^3^Centre for Microscopy, Characterisation and Analysis, University of Western Australia, Perth, WA, Australia

**Keywords:** metaorganism, holobiont, carbon translocation, nitrogen uptake, *Symbiodinium*, selfish symbiont

## Abstract

The symbiosis between cnidarian hosts and microalgae of the genus *Symbiodinium* provides the foundation of coral reefs in oligotrophic waters. Understanding the nutrient-exchange between these partners is key to identifying the fundamental mechanisms behind this symbiosis, yet has proven difficult given the endosymbiotic nature of this relationship. In this study, we investigated the respective contribution of host and symbiont to carbon and nitrogen assimilation in the coral model anemone Aiptaisa. For this, we combined traditional measurements with nanoscale secondary ion mass spectrometry (NanoSIMS) and stable isotope labeling to investigate patterns of nutrient uptake and translocation both at the organismal scale and at the cellular scale. Our results show that the rate of carbon and nitrogen assimilation in Aiptasia depends on the identity of the host and the symbiont. NanoSIMS analysis confirmed that both host and symbiont incorporated carbon and nitrogen into their cells, implying a rapid uptake and cycling of nutrients in this symbiotic relationship. Gross carbon fixation was highest in Aiptasia associated with their native *Symbiodinium* communities. However, differences in fixation rates were only reflected in the δ^13^C enrichment of the cnidarian host, whereas the algal symbiont showed stable enrichment levels regardless of host identity. Thereby, our results point toward a “selfish” character of the cnidarian—*Symbiodinium* association in which both partners directly compete for available resources. Consequently, this symbiosis may be inherently instable and highly susceptible to environmental change. While questions remain regarding the underlying cellular controls of nutrient exchange and the nature of metabolites involved, the approach outlined in this study constitutes a powerful toolset to address these questions.

## Introduction

The ecological success of coral reefs in nutrient poor waters relies on the nutrient-exchange between cnidarians and dinoflagellate algae of the genus *Symbiodinium* living in the host's tissues (Muscatine and Porter, 1977; Falkowski et al., 1984; Hatcher, 1988, 1997). In this association, the endosymbiotic algae translocate the majority of their photosynthetically-fixed carbon to the host, which in turn provides inorganic nutrients from its metabolism to sustain algal productivity (Muscatine, 1967; Muscatine et al., 1981; Falkowski et al., 1984; Rädecker et al., 2017). The efficient recycling of organic as well as inorganic nutrients within this symbiosis underpins the high productivity of coral reefs in the absence of major sources of allochthonous nutrients (Muscatine and Porter, 1977; Wang and Douglas, 1998). Yet, this ecosystem is in global decline as anthropogenic environmental change impedes the role of cnidarians as key ecosystem engineers (Knowlton, 2001; Wild et al., 2011). Mass bleaching events, i.e. the disruption of the cnidarian—*Symbiodinium* symbiosis signified by the expulsion of symbionts and physical whitening of corals on broad scales, are among the dominant drivers of this decline (Bellwood et al., 2004; Hughes et al., 2017). Understanding the causes of this symbiotic breakdown requires considering these symbiotic organisms as holobionts: complex metaorganisms that arise from the interactions of the hosts and their associated microorganisms such as protists, bacteria, and archaea (Rosenberg et al., 2007). A crucial attribute of cnidarian holobionts is the ability to assimilate and recycle nutrients (Suggett et al., 2017). In particular, nitrogen cycling appears to be key to the functioning of these holobionts (Rädecker et al., 2015; Pogoreutz et al., 2017), since the growth of *Symbiodinium* is nitrogen-limited in a stable symbiosis (Muscatine et al., 1989; Belda et al., 1993; Falkowski et al., 1993; Rädecker et al., 2015; Aranda et al., 2016). Nitrogen limitation might stabilize symbiont populations and facilitate the translocation of photosynthates to the host (Ezzat et al., 2015), a process providing most of the energy required for the host's metabolism (Falkowski et al., 1984; Tremblay et al., 2012).

Despite the importance of disentangling the individual contribution of host and symbionts to holobiont nutrient cycling (Yellowlees et al., 2008; Starzak et al., 2014; Leal et al., 2015; Rädecker et al., 2015), studying these processes in scleractinian corals has proven difficult due to the complex and interwoven nature of the coral holobiont. As most corals are associated with a diverse *Symbiodinium* community and are difficult to maintain in a symbiont-free stage (Baker, 2003; Wang et al., 2012), identifying underlying processes within these symbiotic interactions is challenging. In contrast, the emerging model organism Aiptasia (*sensu Exaiptasia pallida;* Grajales and Rodríguez, 2014) promises to be an easy and cost-effective tool to study cnidarian—*Symbiodinium* interactions. While this sea anemone differs from scleractinian corals in some key functional traits, most notably the lack of a calcareous skeleton, it features distinct advantages for the study of cnidarian—*Symbiodinium* symbioses (Voolstra, 2013; Baumgarten et al., 2015; Röthig et al., 2016): (I) it can be reared in clonal lines, enabling the study of processes in the absence of biological variation (Weis et al., 2008); (II) animals can be easily maintained in a symbiont-free stage, allowing the study of host processes in the absence of symbionts (Voolstra, 2013); (III) symbiont-free Aiptasia can be re-infected with specific symbiont strains, enabling the comparison of different symbionts (including those commonly associated with corals) in the same host background *in hospite* (Wolfowicz et al., 2016); (IV) natural populations of Aiptasia can be found in a range of environmental conditions and in association with different symbionts, offering a natural laboratory to study adaptation and coevolution in this symbiosis (Thornhill et al., 2013; Voolstra, 2013); (V) an extensive array of genetic resources is available for Aiptasia, allowing to link genetic and physiological traits (Baumgarten et al., 2015). These distinct advantages will prove especially powerful to study metabolic interactions between host and symbionts when combined with state of the art imaging techniques such as nano-scale secondary ion mass spectrometry (NanoSIMS). Coupled with stable isotope labeling, this technology enables imaging of metabolic processes at subcellular resolution and consequently quantification of nutrient assimilation at the single-cell level for each symbiotic partner (Kopp et al., 2013; Pernice et al., 2014). NanoSIMS has opened doors to an unprecedented level of information across all fields of biology and has previously been successfully applied to corals (Lechene et al., 2007; Pernice et al., 2012, 2014; Kopp et al., 2013; Musat et al., 2016).

In this study, using the combined advantages of the Aiptasia model system and high resolution NanoSIMS, we sought to investigate the relative contribution of cnidarian host identity and associated *Symbiodinium* type to assimilate dissolved inorganic nitrogen and carbon both at the organismal and at the cellular level. By doing this, we aim to promote the use of Aiptasia as a model for the study of metabolic interactions in the cnidarian-*Symbiodinium* symbiosis.

## Materials and methods

### Maintenance of Aiptasia

Four different host–symbiont pairings were maintained in separate batches. These combinations involved two different host clonal lines [CC7 (Sunagawa et al., 2009) and H2 (Xiang et al., 2013)] as well as two different symbiont populations (A4 and B1 dominated; Grawunder et al., 2015). While CC7 Aiptasia can form stable associations with a diversity of *Symbiodinium* types, H2 Aiptasia show high fidelity to their native *Symbiodinium* community suggesting a higher selectivity and/or specificity with their symbionts (Thornhill et al., 2013). This specificity of H2 Aiptasia hinders reinfection with other symbionts thereby preventing a full factorial design in this study. Nevertheless, these host clonal lines provide an ideal basis for the comparison of symbiont diversity and specificity.

**Figure 1:**
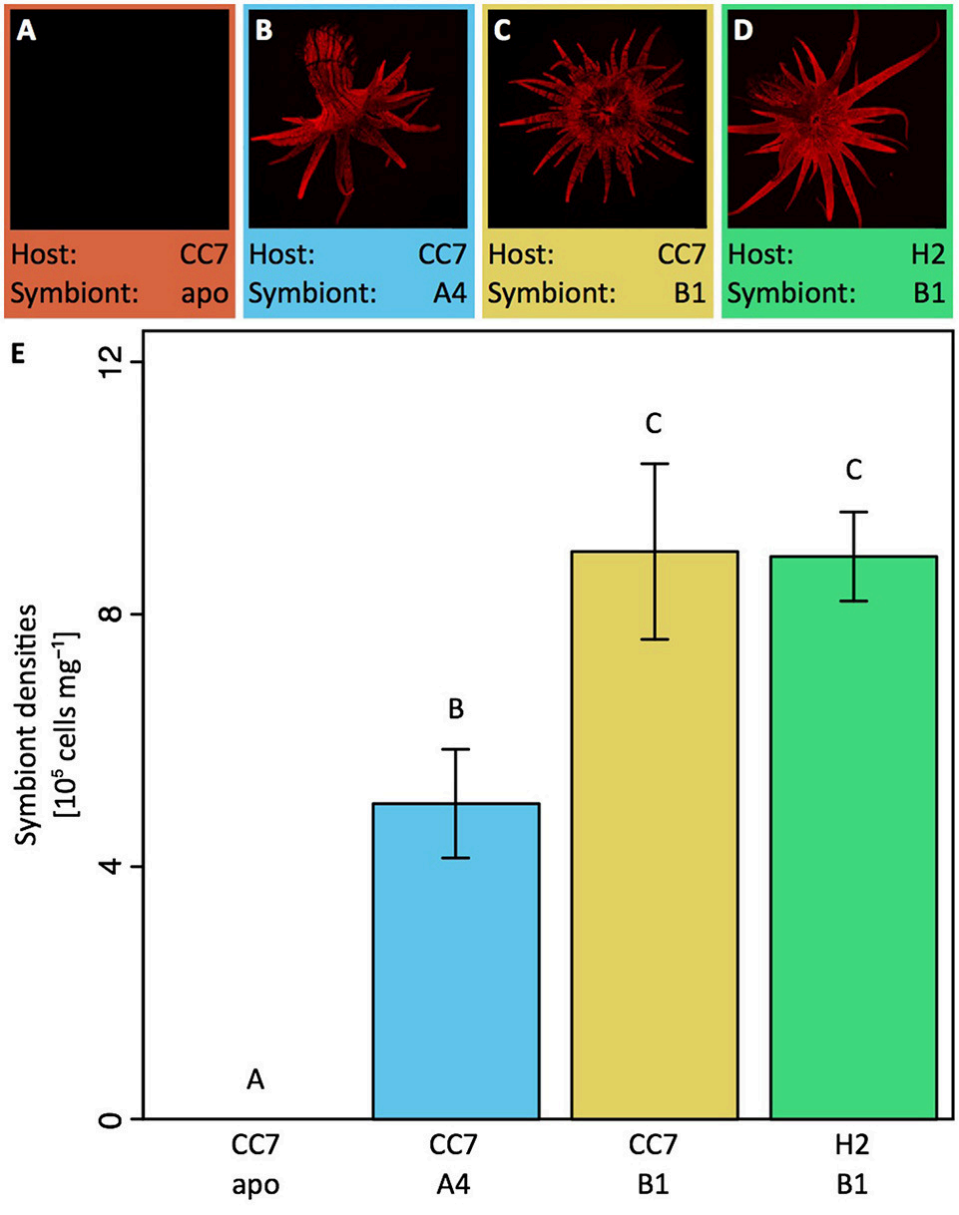
Fluorescence microscopy overview of the four host—symbiont combinations **(A–D)** to visualize *in hospite* chlorophyll autofluorescence of endosymbiotic *Symbiodinium* (diameter of animals ~ 1.5 cm). Notably, symbiont densities **(E)** of these host—symbiont combinations differed between symbiont types but not between hosts harboring the same symbiont when normalized to host protein content. All data are shown as mean ± SE (*n* = 8 animals each). Different letters above bars indicate significant differences between groups (*p* < 0.05). CC7, H2, Aiptasia clonal lines; A4, B1, *Symbiodinium* types; apo, aposymbiotic.

To allow comparison of symbiont types within the same host line and to compare performance of the same symbiont type within different host lines, CC7 Aiptasia were bleached and reinfected with type B1 (strain SSBO1) symbionts, previously isolated from H2 Aiptasia. For this, aposymbiotic CC7 Aiptasia were generated and reinfected as described by Baumgarten et al. (2015). In brief, animals were repeatedly bleached by incubation in 4°C sterile seawater for 4 h, followed by 1–2 days at 25°C in sterile seawater containing the photosynthesis inhibitor diuron. Aposymbiotic animals were maintained for at least 1 month prior to reinfection to confirm absence of residual symbionts. For reinfection, aposymbiotic animals were subjected to three cycles of incubation for 1 day in sterile seawater containing 10^5^ *Symbiodinium* cells mL^−1^ followed by *Artemia salina* nauplii feeding the next day. Thus, the four combinations were: aposymbiotic CC7 Aiptasia, CC7 Aiptasia with its native A4 symbionts; CC7 Aiptasia reinfected with B1 symbionts and H2 Aiptasia with its native B1 symbionts (Figures 1A–D). Animals were reared in autoclaved seawater (35 PSU, 25°C, ~80 μmol photons m^−2^ s^−1^ on a 12:12 h light:dark schedule) and fed with freshly hatched *A. salina* nauplii three times per week. Notably, while these light levels are low compared to shallow coral reef environments, they were chosen to support optimal growth of animals and are in the range of previous studies working with Aiptasia (Muller-Parker, 1984; Lehnert et al., 2014; Hillyer et al., 2016). Animal cultures were propagated under these conditions for more than 1 year to ensure anemones recovered from bleaching and reinfection procedures and to confirm the stability of native and introduced symbiotic associations. Stability of *Symbiodinium* communities was monitored using qPCR as outlined by Correa et al. (Correa et al., 2009). Any feeding was abandoned 3 days prior to measurements to exclude potential confounding effects. Thereby this experimental design allowed us to disentangle the contribution of host and symbionts to holobiont nutrient cycling in three comparisons: (I) between different symbionts within the same host line, (II) between different hosts lines with the same symbiont, and (III) between symbiotic and aposymbiotic states within the same host line.

### Oxygen flux measurements

Net photosynthesis and respiration rates were measured via oxygen (O_2_) evolution and consumption measurements during light and dark incubations, respectively. For this purpose, four specimens of each host–symbiont combination were transferred into 25 ml glass chambers filled with sterile seawater. Specimens were left to settle for 30 min in the dark, before magnetic stirrers were turned on to prevent stratification of the water column. Subsequently, O_2_ concentrations were recorded once per second over the course of 30 min incubations in the light (~80 μmol photons m^−2^ s^−1^, 25°C) and dark (<1 μmol photons m^−2^ s^−1^, 25°C) using FireSting O2 optical oxygen meters (PyroScience, Germany). Following incubation all specimens were immediately flash frozen and stored at −20°C until further analysis. Net photosynthesis (inferred from light incubations) as well as respiration (inferred from dark incubations) rates were corrected for seawater controls and normalized to total protein content and *Symbiodinium* densities of specimens. O_2_ fluxes of net photosynthesis and respiration rates were transformed into their carbon equivalents using the photosynthetic and respiration quotients of 1.1. and 0.9 as proposed by Muscatine et al. (1981). Gross photosynthesis rates (expressed as pmol C symbiont cell^−1^ h^−1^ and μmol C mg host protein^−1^ h^−1^, respectively) were calculated according to:

$$\begin{array}{lll} gross\;photosynthesis\;rate & = & net\;photosynthesis\;rate\\ \;+|respiration\;rate|. \end{array}$$

### Quantification of ${\text{NH}}_{4}^{+}$ uptake and release

Net ammonium (${\text{NH}}_{4}^{+}$) uptake rates were assessed at the holobiont level during light (~80 μmol photons m^−2^ s^−1^, 25°C) and dark (<1 μmol photons m^−2^ s^−1^, 25°C) conditions using the depletion technique (Godinot et al., 2011). Four specimens of each host–symbiont combination were incubated for 60 min in 25 ml chambers filled with ${\text{NH}}_{4}^{+}$-enriched artificial seawater (ASW) with a final concentration of 5 μM (Harrison et al., 1980). Ten milliliters water samples were collected before and after the incubation, filtered (45 μm) and immediately analyzed for ${\text{NH}}_{4}^{+}$ concentrations using an autoanalyzer (SA3000/5000 Chemistry Unit, SKALAR, Netherlands). Differences in ${\text{NH}}_{4}^{+}$ concentrations were corrected for seawater controls and normalized to incubation time, total host protein content and *Symbiodinium* densities of specimens to obtain net uptake rates during both light and dark incubations.

### Protein content, *Symbiodinium* density, and chlorophyll concentrations

Frozen specimens were defrosted in 500 μl sterile saline water and homogenized using a Micro DisTec Homogenizer 125 (Kinematica, Switzerland). An aliquot of the homogenate was immediately analyzed for total protein content as well as symbiont concentrations, respectively. For total host protein content, *Symbiodinium* cells were removed by brief centrifugation and the supernatant was analyzed with the Micro BCA Protein Assay Kit (Thermo Scientific, USA) using 150 μl of 15x diluted tissue slurry as per manufacturer instructions. Likewise, *Symbiodinium* density was quantified by flow cytometry (BD LSRFortessa, BD Biosciences, USA) using 100 μl of strained tissue slurry. Cells were excited at a wavelength of 488 nm and fluorescence emission was recorded at 695/40 nm. *Symbiodinium* cell densities were quantified in triplicate measurements (20 μl each) based on forward-scattered light and chlorophyll autofluorescence signals of recorded events.

### Isotope labeling and sample preparation

To corroborate nitrogen and carbon assimilation rates on the holobiont level, an isotopic labeling experiment was conducted for subsequent nanoscale secondary ion mass spectrometry (NanoSIMS) analysis. Individual specimens of each host–symbiont combination were incubated for 24 h (12:12 h light dark cycle) in 25 ml incubation chambers containing ASW. For isotopic enrichment, freshly prepared ASW, essentially free from bicarbonate and ammonium, was supplemented with NaH^13^CO_3_ (isotopic abundance of 99%) as well as ^15^NH_4_Cl (isotopic abundance of 99%) at a final concentration of 2 mM and 5 μM, respectively (adapted from Harrison et al., 1980). Following incubation, all specimens were immediately transferred to a fixative solution (2.5% glutaraldehyde, 1 M cacodylate) and stored at 4°C until further processing (within 14 days).

Individual tentacles were collected from each anemone under a stereomicroscope for further sample preparation adapted after Pernice et al. (2012) and Kopp et al. (2015). First, samples were post-fixed for 1 h at RT in 1% OsO_4_ on Sörensen phosphate buffer (0.1 M). Samples were dehydrated in a series of increasing ethanol concentrations (50, 70, 90, 100%) followed by 100% acetone. Tissues were then gradually infiltrated with SPURR resin of increasing concentrations (25, 50, 75, 100%). Subsequently, tissues were embedded in SPURR resin and cut into 100 nm sections using an Ultracut E microtome (Leica Microsystems, Germany) and mounted on finder grids for Transmission Electron Microscopy (ProsciTech, Australia).

### NanoSIMS imaging

Gold-coated sections were imaged with the NanoSIMS 50 ion probe at the Center for Microscopy, Characterisation and Analysis at the University of Western Australia. Surfaces of samples were bombarded with a 16 keV primary Cs^+^ beam focused to a spot size of about 100 nm, with a current of ~2 pA. Secondary molecular ions ^12^C^12^C^−^, ^12^C^13^C^−^, ^12^C^14^N-, and ^12^C^15^N^−^ were simultaneously collected in electron multipliers at a mass resolution (M/ΔM) of about 8,000, enough to resolve the ^12^C^13^C^−^ from the ^12^C_2_^1^H^−^ peak and the ^13^C^14^N^−^ and ^12^C^15^N^−^ peaks from one another. Charge compensation was not necessary. Five images of different areas within the gastrodermis of the tentacle (25–45 μm raster with 256 × 256 pixels) were recorded for all targeted secondary molecular ions by rastering the primary beam across the sample with a dwell-time of 10–20 ms per pixel. After drift correction, the ^13^C/^12^C or ^15^N/^14^N maps were expressed as a hue-saturation-intensity image (HSI), where the color scale represents the isotope ratio. Image processing was performed using the ImageJ plugin OpenMIMS (National Resource for Imaging Mass Spectrometry, https://github.com/BWHCNI/OpenMIMS/wiki).

Enrichment of the isotope labels was quantified for 20 Regions of Interest (ROIs) (circles of 2–10 μm) per category (symbiont cells, gastrodermal host tissue and gastrodermal vesicles) for each host–symbiont combination, and expressed using δ^13^C and δ^15^N notation. Gastrodermal host tissue was quantified in the form of ROIs placed adjacent to symbiont cells as clear cell boundaries were not always identifiable.

Unlabeled Aiptasia served as unlabeled controls. δ^13^C and δ^15^N enrichment (expressed in %0) was quantified as follows:

$$\begin{array}{lll} \delta^{13}C & = & \left(\left(\frac{C_{sample}}{C_{unlabelled}}\right)-1\right)\;\times \;10^{3}\text{ and},\\ \delta^{15}N & = & \left(\left(\frac{N_{sample}}{N_{unlabelled}}\right)-1\right)\;\times \;10^{3}, \end{array}$$

where N is the ^15^N/^14^N ratio of sample or unlabeled control and C is the ^13^C/^12^C ratio (measured as ^12^C^13^C^−^/^12^C^12^C^−^ ions) of sample or unlabeled control, respectively. In this context, it is important to note that carbon and nitrogen incorporation at the cellular level was likely underestimated in our study as sample preparation for NanoSIMS may result in partial extraction of biomolecules.

### Statistical analysis

All statistical analyses were conducted with R version 3.2.5 (R Development Core Team, 2015). Data were tested for normal distribution using the Shapiro-Wilk test. All measurements at the holobiont level (symbiont densities, gross photosynthesis, respiration, net ${\text{NH}}_{4}^{+}$ uptake) followed normal distribution and were analyzed with a one-way analysis of variance (ANOVA) using host-symbiont combination as explanatory variable; only gross photosynthesis rates normalized by symbiont density were right-skewed and did not follow a normal distribution and hence were analyzed with a generalized linear model (GLM) using host-symbiont combination as the explanatory variable. Similarly, δ^13^C and δ^15^N enrichment data did not follow a normal distribution and were analyzed in two-factorial GLMs using additive as well as interactive effects of host-symbiont combination as well as holobiont compartment (host, lipid body, symbiont). All GLMs were fitted with Gamma distribution and “log” function to optimize the fit of the model. Fit of model residuals were confirmed using the qqPlot() function as implemented in the “car” package for R (Fox and Weisberg, 2011). An overview of replication and model results is provided in the Supplementary Information Table S1. Adjustment for multiple comparisons between host—symbiont combinations and holobiont compartments was done following the Bonferroni procedure. Significant differences identified via the post hoc comparison are indicated in the figures as different letters above bars.

## Results

### Symbiont densities

Aiptasia of the clonal line CC7 with their native symbiont community (*Symbiodinium* type A4) contained a significantly lower density of symbionts when normalized to host protein content compared to the other two symbiotic host–symbiont combinations (Figure 1E, see Supplementary Information Table S1 for an overview of statistical model results). Notably, symbiont densities in Aiptasia were of the same order of magnitude as previously reported for scleractinian corals (Cunning and Baker, 2014; Ziegler et al., 2015). As expected, no symbionts were detected in aposymbiotic Aiptasia.

### Carbon assimilation and translocation

**Figure 2:**
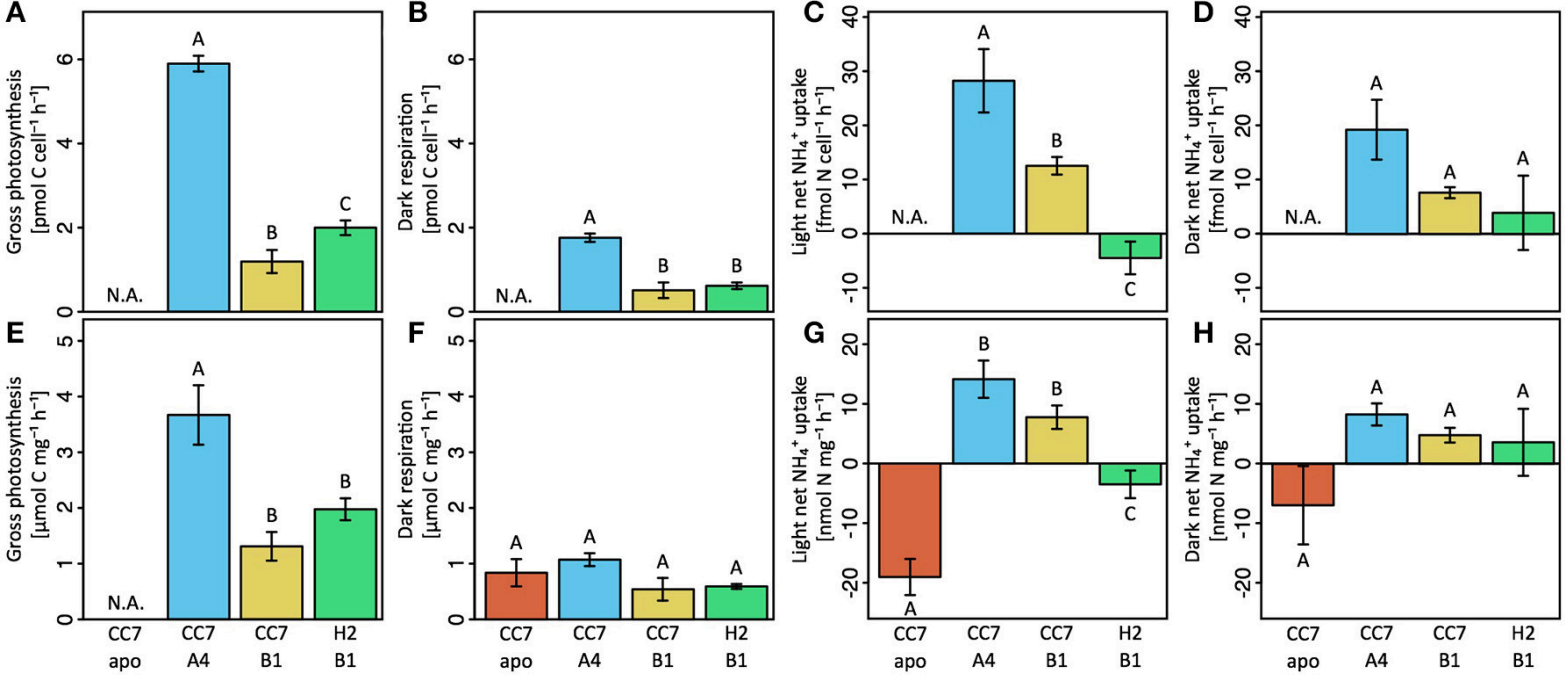
Gross photosynthesis **(A,E)**, dark respiration **(B,F)**, light ${\text{NH}}_{4}^{+}$ uptake **(C,G)** and dark ${\text{NH}}_{4}^{+}$ uptake **(D,H)** rates of Aiptasia were normalized either to symbiont density **(A–D)** or total host protein content **(E–H)**. Gross photosynthesis rates were calculated as the sum of net photosynthesis and respiration rates (P_G_ = P_N_ + |R|). Net ${\text{NH}}_{4}^{+}$ uptake was quantified with the ammonium depletion method. All data shown as mean ± SE (*n* = 4 animals each). Different letters above bars indicate significant differences between groups (*p* < 0.05).

**Figure 3:**
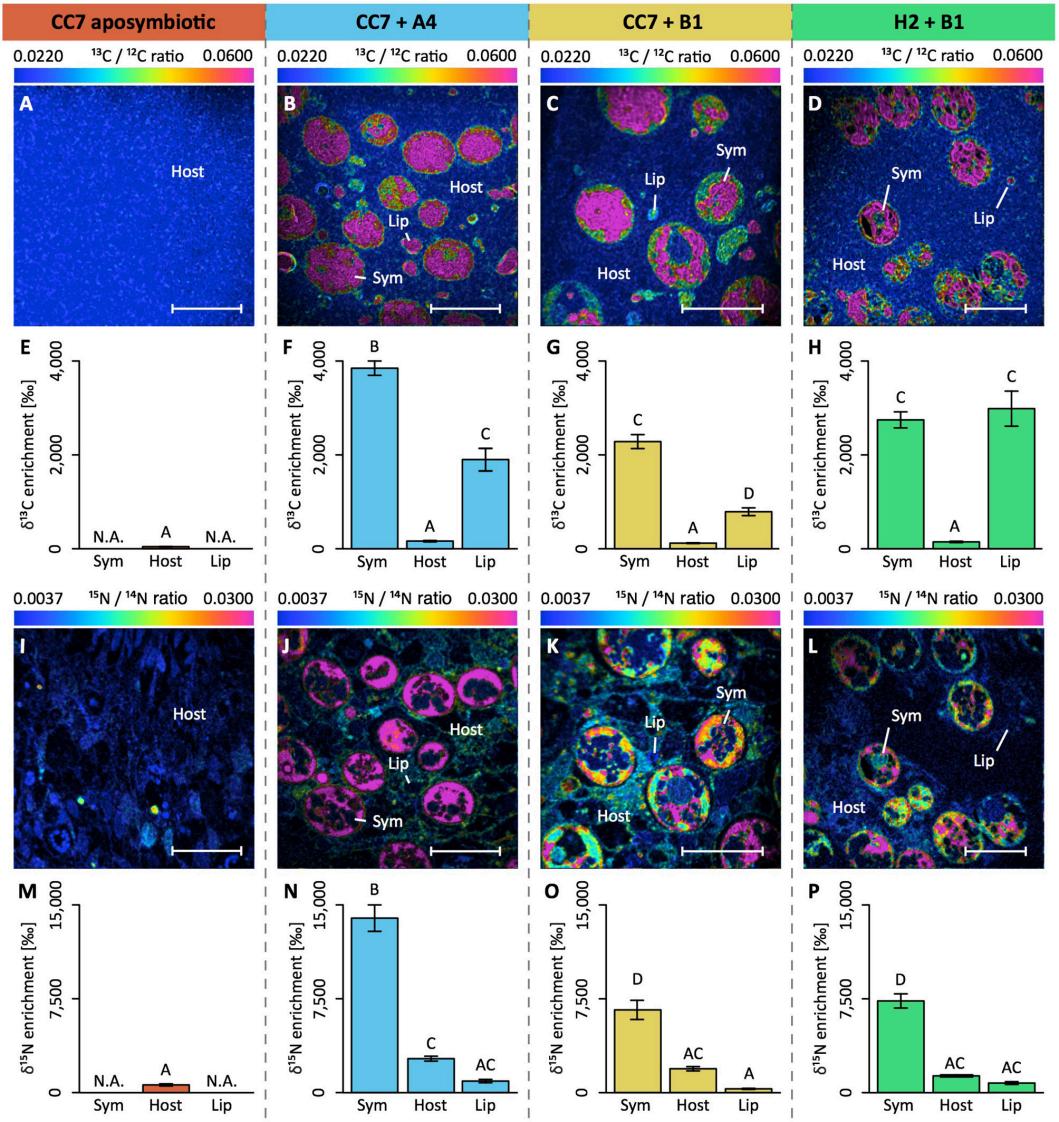
NanoSIMS imaging and quantification of cell-specific carbon (as ^13^C-bicarbonate) and nitrogen (as ^15^N-ammonium) assimilation within the Aiptasia—*Symbiodinium* symbiosis. Representative images of the distribution of ^13^C/^12^C ratio **(A–D)** and of ^15^N/^14^N ratio **(I–L)** within the Aiptasia holobiont are displayed as Hue Saturation Intensity (HSI). The rainbow scale indicates the ^13^C/^12^C and ^15^N/^14^N ratio, respectively. Blue colors indicate natural abundance isotope ratios shifting toward pink with increasing ^13^C and ^15^N incorporation levels, respectively. For each NanoSIMS image, the δ^13^C **(E–H)** and δ^15^N **(M–P)** enrichment were quantified for individual Regions Of Interest (ROIs) that were defined in OpenMIMS by drawing (I) the contours of the symbionts, and circles covering (II) the adjacent host tissue and (III) the host lipid bodies. Scale bars represent 10μm. Sym, *Symbiodinium* cell; Host, tissue (host); Lip, lipid body (host). All data shown as mean ± SE (*n* = 20 ROIs each). Different letters above bars indicate significant differences between groups (*p* < 0.05).

Host–symbiont combinations of Aiptasia showed distinct differences in carbon fixation both at the holobiont (Figure 2) as well as at the cellular level (Figure 3). While fixation rates were highly variable between the three combinations of symbiotic Aiptasia, no carbon fixation was detectable in aposymbiotic Aiptasia, confirming that carbon assimilation was photosynthetically driven. At the holobiont level, gross photosynthesis was highest in Aiptasia of the clonal line CC7 with their native Clade A symbionts after normalization to symbiont cells (Figure 1A) or host protein content (Figure 1E). In contrast, CC7 Aiptasia symbiotic with Clade B (type B1, SSBO1) *Symbiodinium* showed the lowest gross photosynthesis rates of all symbiotic Aiptasia combinations. In particular, rates were lower than H2 Aiptasia hosting the same type B1 dominated symbiont community. Photosynthetic carbon fixation was more than three-fold higher than dark respiratory carbon consumption in all symbiotic Aiptasia groupings. Overall, dark respiration rates largely followed patterns of gross photosynthesis rates when normalized to *Symbiodinium* content, with CC7 Aiptasia symbiotic with type A4 having higher respiration rates than the other two symbiotic Aiptasia combinations hosting type B1 *Symbiodinium*. In contrast, no significant differences in respiration rates were detectable between the fours host—symbiont combinations when normalized to host protein content (Figures 2B,F).

Isotope labeling and NanoSIMS imaging revealed that the observed differences in carbon fixation at the holobiont level translated into an intricate picture at the cellular level (Figures 3A–H). First, δ^13^C enrichment was evident in both host and symbiont cells in all symbiotic Aiptasia groupings (Figures 3B–D). Second, although enrichment was highest in *Symbiodinium* cells, localized regions of <5 μm diameter in the host tissue (referred to as “lipid bodies” from this point on) also showed significantly higher rates of enrichment compared to the surrounding host tissue. Third, Clade B *Symbiodinium* showed no differences in ^13^C-incorporation depending on the host, and incorporation rates were 30–40% lower than in Clade A symbionts. Host lipid bodies, on the contrary, showed a reversed picture with Clade B associated H2 Aiptasia having the highest and Clade B associated CC7 Aiptasia having the lowest ^13^C assimilation rates, despite harboring the same symbiont types.

### ${\text{NH}}_{4}^{+}$ assimilation and release

Similar to carbon fixation, strong differences in ${\text{NH}}_{4}^{+}$ assimilation were evident between the experimental groups of Aiptasia at both holobiont and cellular levels. At the holobiont level, all four host—symbiont combinations showed higher ${\text{NH}}_{4}^{+}$ uptake/release rates during the light (Figures 2C,G), compared to dark conditions (Figures 2D,H). When normalized to host protein content, aposymbiotic Aiptasia showed the highest net release of ${\text{NH}}_{4}^{+}$ at the holobiont level both during light (Figure 2G) and dark incubations (Figure 2H). Albeit significantly lower, symbiotic H2 Aiptasia also had a net release of ${\text{NH}}_{4}^{+}$ into the surrounding seawater during the light incubations, yet took up ${\text{NH}}_{4}^{+}$ during dark incubations. In contrast, both groups of symbiotic CC7 Aiptasia showed a net uptake of ${\text{NH}}_{4}^{+}$ by the holobiont during both light and dark conditions. Further, the uptake rate was affected by the associated symbiont community, with Clade A dominated CC7 holobionts taking up more ${\text{NH}}_{4}^{+}$ than their Clade B infected counterparts (Figures 2C,G).

Although ${\text{NH}}_{4}^{+}$ assimilation ranged from net uptake to net release in the different experimental groups, NanoSIMS imaging confirmed that all four host–symbiont combinations incorporated ^15^N into their cells (Figures 3I–P). While δ^15^N signatures were highest in *Symbiodinium* cells, ^15^N assimilation was also observed within the cnidarian host tissue including that of aposymbiotic Aiptasia. Similar to δ^13^C patterns, δ^15^N enrichment in *Symbiodinium* cells aligned with algal symbiont type rather than host identity, and Clade B symbionts showed lower rates of incorporation than Clade A. Conversely, ^15^N incorporation into host cells was not significantly different between symbiotic Aiptasia groupings, irrespective of their symbiont type. Aposymbiotic CC7 Aiptasia had the lowest overall ^15^N incorporation into their tissue, yet showed small (<5 μm in diameter) and localized regions of high enrichment. In contrast, the afore mentioned lipid bodies of high δ^13^C-enrichment showed consistently lower δ^15^N-signatures than surrounding host tissue in all three symbiotic Aiptasia strains.

## Discussion

Aiptasia has proven to be a powerful emerging tool for the genetic and molecular study of the cnidarian—alga symbiosis (Sunagawa et al., 2009; Thornhill et al., 2013; Voolstra, 2013; Baumgarten et al., 2015; Bellis et al., 2016; Dani et al., 2017; Matthews et al., 2017). Beyond these realms, only a few studies have begun to exploit the advantages Aiptasia has to offer (Tolleter et al., 2013; Starzak et al., 2014; Leal et al., 2015; Biquand et al., 2017; Gegner et al., 2017). Here, we set out to showcase the use of Aiptasia as a model to study nutrient cycling in the cnidarian—alga symbiosis. While NanoSIMS has been successfully used previously to study nutrient uptake in corals (Ceh et al., 2013; Kopp et al., 2015; Lema et al., 2016), the flexibility of the Aiptasia model enabled us to decouple the relative contribution of host and symbionts to nutrient cycling. We could identify differences in nutrient assimilation across different host–symbiont associations, both at the holobiont as well as the cellular level. Yet, only the integration of both levels of biological organization allowed us to comprehensively disentangle some of the intricacies of nutrient cycling in the Aiptasia holobiont.

### Carbon cycling in Aiptasia

All three groups of symbiotic Aiptasia showed high rates of gross photosynthesis that exceeded their respiratory carbon requirements thereby supporting net productivity of the holobiont required for stable symbiotic associations (Muscatine et al., 1981; Muller-Parker, 1984). Yet, differences in gross photosynthesis between host–symbiont combinations were evident at the holobiont level. Gross photosynthesis rates differed between the same host infected with different algal symbionts and between different hosts infected with the same algal symbionts. Thereby our results support the findings by Starzak et al. (2014) who reported differences in carbon flux depending on symbiont type and between heterologous and homologous symbionts in Aiptasia, confirming previous observations that carbon fixation depends on the interaction of both host and symbionts (Goulet et al., 2005; Pernice et al., 2014; Starzak et al., 2014; Leal et al., 2015).

At the cellular level, we observed particular areas of δ^13^C enrichment (hotspots) in the host tissue similar to previous observations (Pernice et al., 2014; Kopp et al., 2016). This high δ^13^C enrichment is further coupled with lower δ^15^N enrichment, suggesting that these hotspots likely constitute a form of carbon storage compartments in the host tissue. Based on shape, size, and location in the tissue, these compartments are most likely lipid bodies (Peng et al., 2011). These cellular organelles are abundant in symbiotic cnidarians as they allow for short-term carbon storage and remobilization depending on cellular carbon availability (Chen et al., 2012). Hence, amount, size and enrichment of these lipid bodies may be an excellent proxy to assess the amount of carbon translocated by *Symbiodinium* to the host, but further studies are needed to unequivocally determine their nature. Lipid body enrichment in the host was highest in H2 Aiptasia and lowest in CC7 Aiptasia, both associated with *Symbiodinium* type B1. Yet, δ^13^C enrichment in algal cells was unaffected by host identity. At the same time, our results revealed that Clade A and B symbionts had distinctly different δ^13^C enrichment, even in the same clonal Aiptasia host line. These differences are likely the consequence of differential metabolic requirements by the specific symbionts. Thus, δ^13^C enrichment may be a powerful tool to differentiate between symbiont types *in hospite*.

Taken together, observed differences in gross carbon fixation at the holobiont level were reflected in the combined δ^13^C enrichment (host tissue + lipid bodies + symbionts) at the cellular level. However, NanoSIMS data revealed that the differences in carbon fixation at the holobiont level were not evenly reflected across all compartments at the cellular level. δ^13^C enrichment in algal cells differed depending on symbiont type (i.e., showed stable δ^13^C enrichment within the same symbiont type), but was unaffected by host identity. In contrast, patterns of carbon fixation rates were directly reflected in the enrichment of the host lipid bodies and host-dependent. Given that both host lineages showed similar respiration rates and symbiont densities when infected with the same symbiont, their differences in host δ^13^C enrichment imply differences in carbon translocation rates. Thereby, this differential enrichment pattern between host and symbionts may have important implications for our understanding of symbiosis functioning. The fact that δ^13^C enrichment in algal cells differed only depending on symbiont type but was unaffected by host identity implies that symbionts retained the same amount of fixed carbon regardless of overall fixed carbon availability. Hence, only excess carbon, not consumed by algal metabolism, appears to be available for translocation to the host. Therefore, factors reducing the availability of excess carbon in the symbiont, may potentially deprive the host of its main energy source, despite harboring viable symbionts in its tissue. This “selfish” aspect of the symbiosis may pose a potential threat to the stability of the holobiont under conditions of reduced fixed-carbon availability, such as those imposed by environmental stress (Anthony et al., 2008, 2009).

### Nitrogen cycling in Aiptasia

The observation of drastically different carbon fixation and translocation rates between different host–symbiont combinations raises questions regarding the underlying regulatory mechanisms of carbon cycling within these symbioses (Suggett et al., 2017). Importantly, nitrogen availability *in hospite* has been proposed to be among the environmental controls of these processes (Wooldridge, 2013; Ezzat et al., 2015; Rädecker et al., 2015; Pogoreutz et al., 2017). Indeed, drastic differences in nitrogen assimilation became evident when comparing different host–symbiont combinations. Strikingly, the two different host lines Aiptasia H2 and CC7 showed net ${\text{NH}}_{4}^{+}$ release and ${\text{NH}}_{4}^{+}$ uptake during the light, respectively, even when hosting the same algal symbionts. These findings suggest that the *in hospite* nutrient availability for the symbiont may be drastically different depending on the associated Aiptasia host. Hence, differences in gross photosynthetic activity and translocation may be partly attributed to variations in availability of nitrogen derived from the host metabolism. Interestingly, while CC7 Aiptasia showed light-enhanced ${\text{NH}}_{4}^{+}$ uptake as previously reported for corals (Grover et al., 2002), H2 Aiptasia showed a net release of ${\text{NH}}_{4}^{+}$ during the light, contrasted by slight uptake during the dark. While we cannot explain the discrepancy at this point, it may reflect differences in internal nitrogen requirement, response times to increased nitrogen availability, and/or uptake efficiency. These differences illustrate the drastic effects of host identity on nitrogen assimilation of the holobiont. At any rate, our results highlight the functional diversity and specificity of cnidarian—*Symbiodinium* symbioses, prompting research across a range of host–symbiont combinations.

In agreement with previous studies, δ^15^N enrichment was highest in *Symbiodinium* cells reflecting their efficient nutrient uptake capacity (Kopp et al., 2013; Pernice et al., 2014; Aranda et al., 2016). However, biomass of the host largely exceeds that of their symbionts. Hence, anemone hosts likely accounted for a large fraction of the nitrogen assimilation in the holobiont, despite having a lower δ^15^N enrichment. At this point it is not possible to distinguish whether the increased δ^15^N enrichment of host tissues in symbiotic animals are due to direct ${\text{NH}}_{4}^{+}$ fixation by the host or the translocation of fixed nitrogen by the symbiont. However, nitrogen assimilation was observed even in the absence of algal symbionts, as evidenced by aposymbiotic Aiptasia. Although these animals showed a high net release of ${\text{NH}}_{4}^{+}$ at the holobiont level, NanoSIMS imaging confirmed the incorporation of ^15^N within localized hotspots of their tissue at low rates. While the exact nature of these hotspots remains unknown at this point, our results confirm that Aiptasia also has the ability to assimilate inorganic nitrogen from seawater as previously reported for corals (Pernice et al., 2012). However, it remains to be determined whether this capability is intricate to the host cellular machinery or a function of associated bacterial symbionts or both (Ceh et al., 2013).

In contrast to δ^15^N enrichment of their hosts, *Symbiodinium* types showed characteristic δ^15^N enrichment patterns regardless of the identity of the host. Hence, δ^15^N enrichment may prove a useful tool to identify symbiont identity *in hospite*, especially when combined with δ^13^C measurements.

Different to carbon fixation measurement, patterns of ${\text{NH}}_{4}^{+}$ uptake on the holobiont level were not directly reflected in the overall δ^15^N enrichment at the cellular level. Specifically, symbiont-free CC7 Aiptasia as well as symbiotic H2 Aiptasia showed net release of ${\text{NH}}_{4}^{+}$ from the holobiont during light conditions, yet NanoSIMS analysis confirmed the incorporation of ^15^N from surrounding seawater. While these differences may be partly attributed to differences in incubation time and light availability for the two measurements, they further suggest that uptake and release of ${\text{NH}}_{4}^{+}$ appear to be in a dynamic equilibrium in Aiptasia. Hence, the stable δ^15^N enrichment of the same symbiont type in CC7 and H2 suggests that the contribution of nitrogen derived from host metabolism was negligible compared to the incorporation of nitrogen from seawater under these conditions. Under natural oligotrophic conditions, however, host metabolism may make a significant contribution to the nitrogen supply of the symbiont.

### Deciphering the role of nutrient cycling in cnidarian holobionts

Our results show (I) that nutrient cycling is drastically altered between symbiotic and aposymbiotic Aiptasia; (II) that different *Symbiodinium* types possess different metabolic capabilities within the same Aiptasia strain and (III) that different Aiptasia strains affect the metabolic performance of the same algal symbiont. Taken together, our findings support the idea of a symbiosis in which partners directly compete for available nutrients and only excess nutrients are exchanged. Consequently, this inherent instability may render this symbiosis highly susceptible to environmental change. Noteworthy, the observed levels and patterns of nutrient assimilation show strong similarities with those previously reported in corals (Grover et al., 2002; Pernice et al., 2012; Kopp et al., 2015), thereby supporting the suitability of Aiptasia as a model for the study of the coral—*Symbiodinium* symbiosis. Although our results require further validation with regard to their wider applicability beyond the Aiptasia model system, our findings showcase the distinct advantages of a model system approach for the study of nutrient cycling in the cnidarian—*Symbiodinium* symbiosis. Nevertheless, questions remain regarding the precise nature of nutrients exchanged in this symbiosis and the underlying processes involved. In particular, future studies should focus on the role of carbon translocation in establishing and maintaining this symbiosis to decipher the intricacies of this symbiosis (Mies et al., 2017). The methodological approach outlined in this study offers a powerful toolset to address such questions. Although optimized to trace carbon and nitrogen assimilation within coral or Aiptasia holobionts (Pernice et al., 2012; Kopp et al., 2015), NanoSIMS can be easily modified depending on the experimental requirements. Specific labeled compounds can also be used as tracers to follow the translocation and uptake of specific molecules in complex systems by coupling the spatial resolution of NanoSIMS with the molecular characterization afforded by time-of-flight secondary ion mass spectrometry (ToF-SIMS) (Raina et al., 2017). As shown here, detailed cellular insights gained from NanoSIMS will prove most powerful when integrated with traditional holobiont based measurements to identify the complexity of processes.

Future research efforts combining a model system approach with field-based coral studies, will help to transform our understanding of the mechanisms underlying this symbiosis and may prompt new solutions to prevent further loss and degradation of reef ecosystems.

## Preprint availability

The original version of the manuscript has been deposited on the bioarxiv.org preprint server (manuscript id: 223933). The manuscript is available under https://www.biorxiv.org/content/early/2017/11/22/223933.

## Author contributions

NR, MA, and CV: conceived and designed the experiment; NR, J-BR, MP, and GP: conducted the experiment; PG and MK: carried out NanoSIMS data acquisition. All authors wrote, revised and approved the manuscript.

### Conflict of interest statement

The authors declare that the research was conducted in the absence of any commercial or financial relationships that could be construed as a potential conflict of interest.

## References

[B1] Anthony K. R. N. Hoogenboom M. O. Maynard J. A. Grottoli A. G. Middlebrook R. (2009). Energetics approach to predicting mortality risk from environmental stress: a case study of coral bleaching. Funct. Ecol. 23, 539–550. 10.1111/j.1365-2435.2008.01531.x

[B2] Anthony K. R. Kline D. I. Diaz-Pulido G. Dove S. Hoegh-Guldberg O. (2008). Ocean acidification causes bleaching and productivity loss in coral reef builders. Proc. Natl. Acad. Sci. U.S.A. 105, 17442–17446. 10.1073/pnas.0804478105 18988740 PMC2580748

[B3] Aranda M. Li Y. Liew Y. J. Baumgarten S. Simakov O. Wilson M.. (2016). Genomes of coral dinoflagellate symbionts highlight evolutionary adaptations conducive to a symbiotic lifestyle. Sci. Rep. 6:39734. 10.1038/srep39734 28004835 PMC5177918

[B4] Baker A. C. (2003). Flexibility and specificity in coral-algal symbiosis: diversity, ecology, and biogeography of *Symbiodinium*. Annu. Rev. Ecol. Evol. Syst. 34, 661–689. 10.1146/annurev.ecolsys.34.011802.132417

[B5] Baumgarten S. Simakov O. Esherick L. Y. Jin Y. Lehnert E. M. Michell C. T.. (2015). The genome of *Aiptasia*, a sea anemone model for coral symbiosis. Proc. Natl. Acad. Sci. U.S.A. 112, 11893–11898. 10.1073/pnas.1513318112 26324906 PMC4586855

[B6] Belda C. A. Lucas J. S. Yellowlees D. (1993). Effects of nutrient supplements on growth of the symbiotic partners. Mar. Biol. 664, 655–664. 10.1007/BF00349778

[B7] Bellis E. S. Howe D. K. Denver D. R. (2016). Genome-wide polymorphism and signatures of selection in the symbiotic sea anemone *Aiptasia*. BMC Genomics 17:160. 10.1186/s12864-016-2488-6 26926343 PMC4772690

[B8] Bellwood D. R. Hughes T. P. Folke C. Nyström M. (2004). Confronting the coral reef crisis. Nature 429, 827–833. 10.1038/nature02691 15215854

[B9] Biquand E. Okubo N. Aihara Y. Rolland V. Hayward D. C. Hatta M.. (2017). Acceptable symbiont cell size differs among cnidarian species and may limit symbiont diversity. ISME J. 11, 1702–1712. 10.1038/ismej.2017.17 28323278 PMC5520142

[B10] Ceh J. Kilburn M. R. Cliff J. B. Raina J. B. van Keulen M. Bourne D. G. (2013). Nutrient cycling in early coral life stages: *Pocillopora damicornis* larvae provide their algal symbiont (*Symbiodinium*) with nitrogen acquired from bacterial associates. Ecol. Evol. 3, 2393–2400. 10.1002/ece3.642

[B11] Chen W. N. U. Kang H. J. Weis V. M. Mayfield A. B. Jiang P. L. Fang L. S. (2012). Diel rhythmicity of lipid-body formation in a coral-*Symbiodinium* endosymbiosis. Coral Reefs 31, 521–534. 10.1007/s00338-011-0868-6

[B12] Correa A. M. S. McDonald M. D. Baker A. C. (2009). Development of clade-specific *Symbiodinium* primers for quantitative PCR (qPCR) and their application to detecting clade D symbionts in Caribbean corals. Mar. Biol. 156, 2403–2411. 10.1007/s00227-009-1263-5

[B13] Cunning R. Baker A. C. (2014). Not just who, but how many: the importance of partner abundance in reef coral symbioses. Front. Microbiol. 5:400. 10.3389/fmicb.2014.00400 25136339 PMC4120693

[B14] Dani V. Priouzeau F. Mertz M. Mondin M. Pagnotta S. Lacas-Gervais S.. (2017). Expression patterns of sterol transporters NPC1 and NPC2 in the cnidarian–dinoflagellate symbiosis. Cell. Microbiol. 19, 1–13. 10.1111/cmi.12753 28544363

[B15] Ezzat L. Maguer J. F. Grover R. Ferrier-Pagès C. (2015). New insights into carbon acquisition and exchanges within the coral – dinoflagellate symbiosis under NH4+ and NO3- supply. Proc. R. Soc. B Biol. Sci. 282:20150610. 10.1098/rspb.2015.0610 26203006 PMC4528508

[B16] Falkowski P. G. Dubinsky Z. Muscatine L. McCloskey L. (1993). Population control in symbiotic corals. Bioscience 43, 606–611. 10.2307/1312147

[B17] Falkowski P. P. G. Dubinsky Z. Muscateine L. Porter J. J. W. (1984). Light and bioenergetics of a symbiotic coral. Bioscience 34, 705–709. 10.2307/1309663

[B18] Fox J. Weisberg S. (2011). An {R} Companion to Applied Regression, 2nd Edn. Thousand Oaks, CA: Sage

[B19] Gegner H. M. Ziegler M. Rädecker N. Buitrago-López C. Aranda M. Voolstra C. R. (2017). High salinity conveys thermotolerance in the coral model Aiptasia. Biol. Open 6, 1943–1948. 10.1242/bio.028878 29175860 PMC5769654

[B20] Godinot C. Grover R. Allemand D. Ferrier-Pagès C. (2011). High phosphate uptake requirements of the scleractinian coral *Stylophora pistillata*. J. Exp. Biol. 214, 2749–2754. 10.1242/jeb.054239 21795572

[B21] Goulet T. L. Cook C. B. Goulet D. (2005). Effect of short-term exposure to elevated temperatures and light levels on photosynthesis of different host-symbiont combinations in the *Aiptasia pallida-Symbiodinium* symbiosis. Limnol. Oceanogr. 50, 1490–1498. 10.4319/lo.2005.50.5.1490

[B22] Grajales A. Rodríguez E. (2014). Morphological revision of the genus *Aiptasia* and the family Aiptasiidae (*Cnidaria, Actiniaria, Metridioidea*). Zootaxa 3826, 55–100. 10.11646/zootaxa.3826.1.2 24990039

[B23] Grawunder D. Hambleton E. A. Bucher M. Wolfowicz I. Bechtoldt N. Guse A. (2015). Induction of gametogenesis in the cnidarian endosymbiosis model *Aiptasia* sp. Sci. Rep. 5:15677. 10.1038/srep15677 26498008 PMC4620495

[B24] Grover R. Maguer J. F. Reynaud-vaganay S. Ferrier-Pages C. (2002). Uptake of ammonium by the scleractinian coral *Stylophora pistillata*: effect of feeding, light, and ammonium concentrations. Limnol. Oceanogr. 47, 782–790. 10.4319/lo.2002.47.3.0782

[B25] Harrison P. J. Waters R. E. Taylor F. J. R. (1980). A broad spectrum artificial sea water medium for coastal and open ocean phytoplankton. J. Phycol. 16, 28–35. 10.1111/j.1529-8817.1980.tb00724.x

[B26] Hatcher B. G. (1988). Coral reef primary productivity: a beggar's banquet. Trends Ecol. Evol. 3, 106–111. 21227159 10.1016/0169-5347(88)90117-6

[B27] Hatcher B. G. (1997). Coral reef ecosystems: how much greater is the whole than the sum of the parts? Coral Reefs 16, 77–91. 10.1007/s003380050244

[B28] Hillyer K. E. Tumanov S. Villas-Bôas S. Davy S. K. (2016). Metabolite profiling of symbiont and host during thermal stress and bleaching in a model cnidarian-dinoflagellate symbiosis. J. Exp. Biol. 219, 516–527. 10.1242/jeb.128660 26685173

[B29] Hughes T. P. Kerry J. Álvarez-Noriega M. Álvarez-Romero J. Anderson K. Baird A.. (2017). Global warming and recurrent mass bleaching of corals. Nature 543, 373–377. 10.1038/nature21707 28300113

[B30] Knowlton N. (2001). The future of coral reefs. Proc. Natl. Acad. Sci. U.S.A. 98, 5419–5425. 10.1073/pnas.091092998 11344288 PMC33228

[B31] Kopp C. Domart-Coulon I. Barthelemy D. Meibom A. (2016). Nutritional input from dinoflagellate symbionts in reef-building corals is minimal during planula larval life stage. Sci. Adv. 2:e1500681. 10.1126/sciadv.1500681 27051861 PMC4820372

[B32] Kopp C. Domart-Coulon I. Escrig S. Humbel B. M. Hignette M. Meibom A. (2015). Subcellular investigation of photosynthesis-driven carbon and nitrogen assimilation and utilization in the symbiotic reef coral *Pocillopora damicornis*. mBio 6:e02299–14. 10.1128/mBio.02299-14 25670779 PMC4337570

[B33] Kopp C. Pernice M. Domart-Coulon I. Djediat C. Spangenberg J. E. Alexander D.. (2013). Highly dynamic cellular-level response of symbiotic coral to a sudden increase in environmental nitrogen. mBio 4:e00052–13. 10.1128/mBio.00052-13 23674611 PMC3656441

[B34] Leal M. C. Hoadley K. Pettay D. T. Grajales A. Calado R. Warner M. E. (2015). Symbiont type influences trophic plasticity of a model cnidarian-dinoflagellate symbiosis. J. Exp. Biol. 218, 858–863. 10.1242/jeb.115519 25617454

[B35] Lechene C. P. Luyten Y. McMahon G. Distel D. L. (2007). Quantitative imaging of nitrogen fixation by individual bacteria within animal cells. Science 317, 1563–1566. 10.1126/science.1145557 17872448

[B36] Lehnert E. M. Mouchka M. E. Burriesci M. S. Gallo N. D. Schwarz J. A. Pringle J. R. (2014). Extensive differences in gene expression between symbiotic and aposymbiotic cnidarians. G3 4, 277–295. 10.1534/g3.113.009084 24368779 PMC3931562

[B37] Lema K. A. Clode P. L. Kilburn M. R. Thornton R. Willis B. L. Bourne D. G. (2016). Imaging the uptake of nitrogen-fixing bacteria into larvae of the coral *Acropora millepora*. ISME J. 10, 18084–11808. 10.1038/ismej.2015.229 26696324 PMC4918436

[B38] Matthews J. L. Crowder C. M. Oakley C. A. Lutz A. Roessner U. Meyer E.. (2017). Optimal nutrient exchange and immune responses operate in partner specificity in the cnidarian-dinoflagellate symbiosis. Proc. Natl. Acad. Sci. U.S.A. 114, 13194–13199. 10.1073/pnas.1710733114 29158383 PMC5740609

[B39] Mies M. Sumida P. Y. G. Rädecker N. Voolstra C. R. (2017). Marine invertebrate larvae associated with *Symbiodinium*: a mutualism from the start? Front. Ecol. Evol. 5:56 10.3389/fevo.2017.00056

[B40] Muller-Parker G. (1984). Photosynthesis-irradiance responses and photosynthetic periodicity in the sea anemone *Aiptasia pulchella* and its zooxanthellae. Mar. Biol. 82, 225–232. 10.1007/BF00392403

[B41] Musat N. Musat F. Weber P. K. Pett-Ridge J. (2016). Tracking microbial interactions with NanoSIMS. Curr. Opin. Biotechnol. 41, 114–121. 10.1016/j.copbio.2016.06.007 27419912

[B42] Muscatine L. (1967). Glycerol excretion by symbiotic algae from corals and *Tridacna* and its control by the host. Science 156, 516–519. 10.1126/science.156.3774.516 17730744

[B43] Muscatine L. Falkowski P. G. Dubinsky P. A. Cook C. A. McCloskey L. R. R. Falkowsk P. G. (1989). The effect of external nutrient resources on the population dynamics of zooxanthellae in a reef coral. Proc. R. Soc. Lond. Ser. B Biol. Sci. 236, 311–324. 10.1098/rspb.1989.0025

[B44] Muscatine L. McCloskey L. R. Marian R. E. (1981). Estimating the daily contribution of carbon from zooxanthellae to coral animal respiration. Limnol. Oceanogr. 26, 601–611. 10.4319/lo.1981.26.4.0601

[B45] Muscatine L. Porter J. W. (1977). Reef corals: mutualistic symbioses adapted to nutrient-poor environments. Bioscience 27, 454–460. 10.2307/1297526

[B46] Peng S. E. Chen W. N. Chen H. K. Lu C. Y. Mayfield A. B. Fang L. S.. (2011). Lipid bodies in coral-dinoflagellate endosymbiosis: proteomic and ultrastructural studies. Proteomics 11, 3540–3555. 10.1002/pmic.201000552 21751349

[B47] Pernice M. Dunn S. R. Tonk L. Dove S. Domart-Coulon I. Hoppe P.. (2014). A nanoscale secondary ion mass spectrometry study of dinoflagellate functional diversity in reef-building corals. Environ. Microbiol. 17, 3570–3580. 10.1111/1462-2920.12518 24902979

[B48] Pernice M. Meibom A. Van Den Heuvel A. Kopp C. Domart-Coulon I. Hoegh-Guldberg O.. (2012). A single-cell view of ammonium assimilation in coral-dinoflagellate symbiosis. ISME J. 6, 1314–1324. 10.1038/ismej.2011.196 22222466 PMC3379633

[B49] Pogoreutz C. Rädecker N. Cárdenas A. Gärdes A. Voolstra C. R. Wild C. (2017). Sugar enrichment provides evidence for a role of nitrogen fixation in coral bleaching. Glob. Chang. Biol. 23, 3838–3848. 10.1111/gcb.13695 28429531

[B50] Rädecker N. Pogoreutz C. Voolstra C. R. Wiedenmann J. Wild C. (2015). Nitrogen cycling in corals: the key to understanding holobiont functioning? Trends Microbiol. 23, 490–497. 10.1016/j.tim.2015.03.008 25868684

[B51] Rädecker N. Pogoreutz C. Wild C. Voolstra C. R. (2017). Stimulated respiration and net photosynthesis in *Cassiopeia* sp. during glucose enrichment suggests in hospite CO2 limitation of algal endosymbionts. Front. Mar. Sci. 4:267 10.3389/fmars.2017.00267

[B52] Raina J. B. Clode P. Cheong S. Bougoure J. Kilburn M. R. Reeder A.. (2017). Subcellular tracking reveals the location of dimethylsulfoniopropionate in microalgae and visualises its uptake by marine bacteria. Elife 6:e23008. 10.7554/eLife.23008 28371617 PMC5380433

[B53] R Development Core Team R. Team R. C. (2015). R: A Language and Environment for Statistical Computing. Vienna: R Foundation for Statistical Computing.

[B54] Rosenberg E. Koren O. Reshef L. Efrony R. Zilber-Rosenberg I. (2007). The role of microorganisms in coral health, disease and evolution. Nat. Rev. Microbiol. 5, 355–362. 10.1038/nrmicro1635 17384666

[B55] Röthig T. Costa R. M. Simona F. Baumgarten S. Torres A. F. Radhakrishnan A. (2016). Distinct bacterial communities associated with the coral model *Aiptasia* in aposymbiotic and symbiotic states with *Symbiodinium*. Front. Mar. Sci. 3:234 10.3389/fmars.2016.00234

[B56] Starzak D. E. Quinnell R. G. Nitschke M. R. Davy S. K. (2014). The influence of symbiont type on photosynthetic carbon flux in a model cnidarian-dinoflagellate symbiosis. Mar. Biol. 161, 711–724. 10.1007/s00227-013-2372-8

[B57] Suggett D. J. Warner M. E. Leggat W. (2017). Symbiotic dinoflagellate functional diversity mediates coral survival under ecological crisis. Trends Ecol. Evol. 32, 735–745. 10.1016/j.tree.2017.07.013 28843439

[B58] Sunagawa S. Wilson E. C. Thaler M. Smith M. L. Caruso C. Pringle J. R.. (2009). Generation and analysis of transcriptomic resources for a model system on the rise: the sea anemone *Aiptasia pallida* and its dinoflagellate endosymbiont. BMC Genomics 10:258. 10.1186/1471-2164-10-258 19500365 PMC2702317

[B59] Thornhill D. J. Xiang Y. Pettay D. T. Zhong M. Santos S. R. (2013). Population genetic data of a model symbiotic cnidarian system reveal remarkable symbiotic specificity and vectored introductions across ocean basins. Mol. Ecol. 22, 4499–4515. 10.1111/mec.12416 23980764

[B60] Tolleter D. Seneca F. O. Denofrio J. C. Krediet C. J. Palumbi S. R. Pringle J. R.. (2013). Coral bleaching independent of photosynthetic activity. Curr. Biol. 23, 1782–1786. 10.1016/j.cub.2013.07.041 24012312

[B61] Tremblay P. Grover R. Maguer J. F. Legendre L. Ferrier-Pagès C. (2012). Autotrophic carbon budget in coral tissue: a new 13C-based model of photosynthate translocation. J. Exp. Biol. 215, 1384–1393. 10.1242/jeb.065201 22442377

[B62] Voolstra C. R. (2013). A journey into the wild of the cnidarian model system *Aiptasia* and its symbionts. Mol. Ecol. 22, 4366–4368. 10.1111/mec.12464 24137737

[B63] Wang J. T. Chen Y. Y. Tew K. S. Meng P. J. Chen C. A. (2012). Physiological and biochemical performances of menthol-induced aposymbiotic corals. PLoS ONE 7:e46406. 10.1371/journal.pone.0046406 23029512 PMC3459915

[B64] Wang J. Douglas A. E. (1998). Nitrogen recycling or nitrogen conservation in an alga-invertebrate symbiosis? J. Exp. Biol. 201, 2445–2453. 9679106 10.1242/jeb.201.16.2445

[B65] Weis V. M. Davy S. K. Hoegh-Guldberg O. Rodriguez-Lanetty M. Pringle J. R. (2008). Cell biology in model systems as the key to understanding corals. Trends Ecol. Evol. 23, 369–376. 10.1016/j.tree.2008.03.004 18501991

[B66] Wild C. Hoegh-Guldberg O. Naumann M. S. Colombo-Pallotta F. Ateweberhan M. Fitt W. K. (2011). Climate change impedes scleractinian corals as primary reef ecosystem engineers. Mar. Freshw. Res. 62, 205–215. 10.1071/MF10254

[B67] Wolfowicz I. Baumgarten S. Voss P. A. Hambleton E. A. Voolstra C. R. Hatta M.. (2016). *Aiptasia* sp. larvae as a model to reveal mechanisms of symbiont selection in cnidarians. Sci. Rep. 6:32366. 10.1038/srep32366 27582179 PMC5007887

[B68] Wooldridge S. A. (2013). Breakdown of the coral-algae symbiosis: towards formalising a linkage between warm-water bleaching thresholds and the growth rate of the intracellular zooxanthellae. Biogeosciences 10, 1647–1658. 10.5194/bg-10-1647-2013

[B69] Xiang T. Hambleton E. A. Denofrio J. C. Pringle J. R. Grossman A. R. (2013). Isolation of clonal axenic strains of the symbiotic dinoflagellate *Symbiodinium* and their growth and host specificity. J. Phycol. 49, 447–458. 10.1111/jpy.12055 27007034

[B70] Yellowlees D. Rees T. A. Leggat W. (2008). Metabolic interactions between algal symbionts and invertebrate hosts. Plant. Cell Environ. 31, 679–694. 10.1111/j.1365-3040.2008.01802.x 18315536

[B71] Ziegler M. Roder C. M. Büchel C. Voolstra C. R. (2015). Mesophotic coral depth acclimatization is a function of host-specific symbiont physiology. Front. Mar. Sci. 2:4 10.3389/fmars.2015.00004

