# Corrigendum: Using Aiptasia as a Model to Study Metabolic Interactions in Cnidarian-*Symbiodinium* Symbioses

**DOI:** 10.3389/fphys.2018.00449

**Published:** 2018-05-15

**Authors:** Nils Rädecker, Jean-Baptiste Raina, Mathieu Pernice, Gabriela Perna, Paul Guagliardo, Matt R. Kilburn, Manuel Aranda, Christian R. Voolstra

**Affiliations:** ^1^Red Sea Research Center, Division of Biological and Environmental Science and Engineering (BESE), King Abdullah University of Science and Technology (KAUST), Thuwal, Saudi Arabia; ^2^Climate Change Cluster, University of Technology Sydney, Sydney, NSW, Australia; ^3^Centre for Microscopy, Characterisation and Analysis, University of Western Australia, Perth, WA, Australia

**Keywords:** metaorganism, holobiont, carbon translocation, nitrogen uptake, *Symbiodinium*, selfish symbiont

During submission of the final version of the manuscript for publication, a previous version of Figure 3 was accidentally uploaded. The labeling of this previous Figure version does not match the annotation in the figure legend. The correct version of Figure 3 and its legend appear below. The authors sincerely apologize for the error. This error does not change the scientific conclusions of the research article.

**Figure 3:**
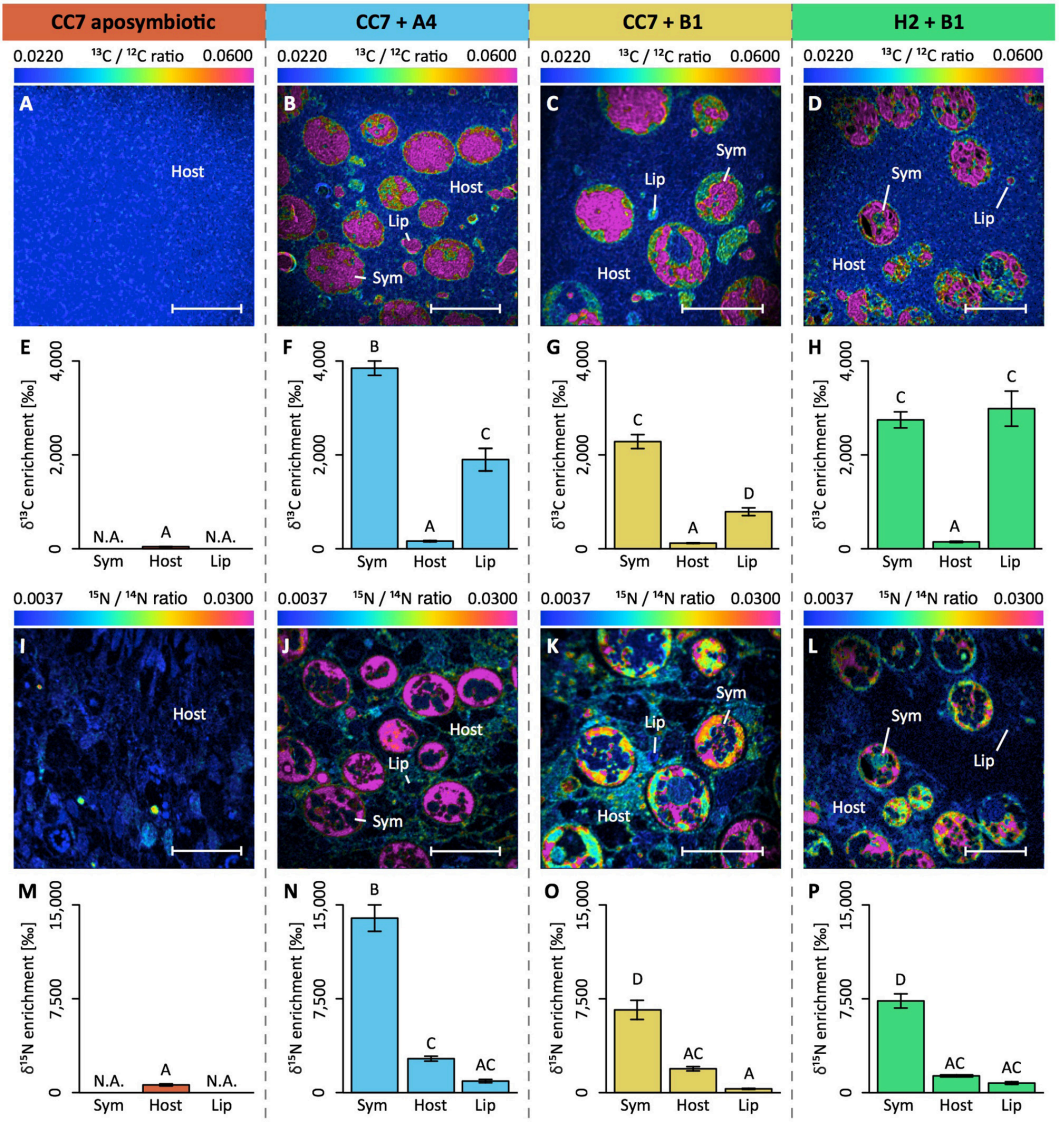
NanoSIMS imaging and quantification of cell-specific carbon (as ^13^C-bicarbonate) and nitrogen (as ^15^N-ammonium) assimilation within the Aiptasia–*Symbiodinium* symbiosis. Representative images of the distribution of ^13^C/^12^C ratio **(A–D)** and of ^15^N/^14^N ratio **(I–L)** within the Aiptasia holobiont are displayed as Hue Saturation Intensity (HSI). The rainbow scale indicates the ^13^C/^12^C and ^15^N/^14^N ratio, respectively. Blue colors indicate natural abundance isotope ratios shifting toward pink with increasing ^13^C and ^15^N incorporation levels, respectively. For each NanoSIMS image, the δ^13^C **(E–H)** and δ^15^N **(M–P)** enrichment were quantified for individual Regions Of Interest (ROIs) that were defined in OpenMIMS by drawing (I) the contours of the symbionts, and circles covering (II) the adjacent host tissue and (III) the host lipid bodies. Scale bars represent 10 μm. Sym, *Symbiodinium* cell; Host, tissue (host); Lip, lipid body (host). All data shown as mean ± SE (*n* = 20 ROIs each). Different letters above bars indicate significant differences between groups (*p* < 0.05).

The original article has been updated.

## Conflict of interest statement

The authors declare that the research was conducted in the absence of any commercial or financial relationships that could be construed as a potential conflict of interest.

