# Supplementary material for: Using Aiptasia as a Model to Study Metabolic Interactions in Cnidarian-Symbiodinium Symbioses
Source: Front Physiol. 2018 Mar 16;9:214. doi: 10.3389/fphys.2018.00214 (PMC5864895; doi:10.3389/fphys.2018.00214)
Supplement: Supplementary file 1 [file Table1.DOCX]

**Table S1** Overview of model results for statistical analysis of individual response parameters. Symbiont/protein ratio **(A),** Gross photosynthesis per protein **(C)**, respiration **(F,G)** as well as ammonium (NH_4_^+^) uptake **(D,E,G,I)** rates were analyzed in one-way analysis of variance (ANOVA) using host—symbiont combination as the dependent variable. Gross photosynthesis per symbiont **(B)** was analyzed in a generalized linear model (GLM) with γ-distribution using host—symbiont combination as the dependent variable, since data did not adhere to a normal distribution. Likewise, 𝛿^13^C and 𝛿^15^N enrichment patterns **(J,K)** were analyzed in two-factorial GLMs with γ-distribution using additive as well as interactive effects of host-symbiont combination and the holobiont compartment.

|  |  |  |  |  |
| --- | --- | --- | --- | --- |
| **A** – **Symbiont/protein ratio** (ANOVA) | *n* | *Df* | *F* | *p* |
| Host—symbiont combination | 32 | 3 | 22.75 | **<0.001** |
|  |  |  |  |  |
|  |  |  |  |  |
| **B** - **Gross photosynthesis per symbiont** (GLM, γ-distr.) | *n* | *Df* | *𝜒^2^* | *p* |
| Host—symbiont combination | 12 | 2 | 66.72 | **<0.001** |
|  |  |  |  |  |
|  |  |  |  |  |
| **C** - **Gross photosynthesis per protein** (ANOVA) | *n* | *Df* | *F* | *p* |
| Host—symbiont combination | 12 | 2 | 11.38 | **0.003** |
|  |  |  |  |  |
|  |  |  |  |  |
| **D – Light net NH_4_^+^ uptake per symbiont** (ANOVA) | *n* | *Df* | *F* | *p* |
| Host—symbiont combination | 12 | 2 | 17.49 | **<0.001** |
|  |  |  |  |  |
|  |  |  |  |  |
| **E – Light net NH_4_^+^ uptake per protein** (ANOVA) | *n* | *Df* | *F* | *p* |
| Host—symbiont combination | 16 | 3 | 30.04 | **<0.001** |
|  |  |  |  |  |
|  |  |  |  |  |
| **F – Dark respiration per symbiont** (ANOVA) | *n* | *Df* | *F* | *p* |
| Host—symbiont combination | 12 | 2 | 28.55 | **<0.001** |
|  |  |  |  |  |
|  |  |  |  |  |
| **G – Dark respiration per protein** (ANOVA) | *n* | *Df* | *F* | *p* |
| Host—symbiont combination | 16 | 3 | 2.05 | 0.160 |
|  |  |  |  |  |
|  |  |  |  |  |
| **H – Dark net NH_4_^+^ uptake per symbiont** (ANOVA) | *n* | *Df* | *F* | *p* |
| Host—symbiont combination | 12 | 2 | 2.44 | 0.142 |
|  |  |  |  |  |
|  |  |  |  |  |
| **I – Dark net NH_4_^+^ uptake per protein** (ANOVA) | *n* | *Df* | *F* | *p* |
| Host—symbiont combination | 16 | 3 | 2.16 | 0.146 |
|  |  |  |  |  |
|  |  |  |  |  |
| **J – 𝛿^13^C enrichment** (GLM, γ-distr.) | *n* | *Df* | *𝜒^2^* | *p* |
| Host—symbiont combination | 200 | 3 | 1252.69 | **<0.001** |
| Compartment (host, symbiont, vesicle) | 200 | 2 | 169.50 | **<0.001** |
| Host—symbiont combination x compartment | 200 | 4 | 54.22 | **<0.001** |
|  |  |  |  |  |
|  |  |  |  |  |
| **K – 𝛿^15^N enrichment** (GLM, γ-distr.) | *n* | *Df* | *𝜒^2^* | *p* |
| Host—symbiont combination | 200 | 3 | 131.39 | **<0.001** |
| Compartment (host, symbiont, vesicle) | 200 | 2 | 782.53 | **<0.001** |
| Host—symbiont combination x compartment | 200 | 4 | 34.11 | **<0.001** |
|  |  |  |  |  |
